# Sexual Dimorphism in Flight-Related Structures of a Paper Wasp

**DOI:** 10.3390/insects17080794

**Published:** 2026-07-31

**Authors:** Bruno Matheus Gomes, André Rodrigues de Souza, Fábio Santos do Nascimento, Carlos E. Sarmiento

**Affiliations:** 1 Departamento de Biologia, Faculdade de Filosofia Ciências e Letras de Ribeirão Preto, Universidade de São Paulo, Bandeirantes, 3900, Ribeirão Preto 14040-901, SP, Brazil; bruno.matheus.gomes@usp.br (B.M.G.);; 2 Departamento de Biologia Geral, Universidade Federal de Viçosa, Avenida Peter Henry Rolfs, s/n, Campus Universitário, Viçosa 36570-900, MG, Brazil; andre.r.souza@ufv.br; 3 Universidad Nacional de Colombia, Sede Bogotá, Facultad de Ciencias, Instituto de Ciencias Naturales, Laboratorio de Sistemática y Biología Comparada de Insectos, Bogotá 11321, CP, Colombia

**Keywords:** allometry, geometric morphometrics, aerodynamics, Vespidae, Hymenoptera

## Abstract

Male and female paper wasps (Vespidae: Polistinae) perform different behavioral roles. Thus, sexes may experience distinct selective pressures that may ultimately affect body size, shape, and proportions. We investigated this in the neotropical *Polistes simillimus*. We compared 100 males and 100 females using linear morphometrics, geometric morphometrics, and allometric analyses of head, mesosoma, forewing, and fore femur. Females were larger than males in all linear measurements. After correcting for body size, males exhibited wider heads, longer mesosomas, and longer fore femora, whereas females had longer heads. Positive allometry of mesosoma length was detected in males and negative allometry of fore femur length in females. Geometric morphometrics confirmed differences in both forewing (rounder in males, elongated in females) and mesosoma (stouter in males). These findings are consistent with the contrasting behavioral demands experienced by each sex; they also generate testable hypotheses regarding the evolution of flight performance in these insects.

## Simple Summary

Given the distinct behavioral roles performed by each sex in social wasps, differences in body size and shape are expected to evolve. We compared morphometric traits in a hundred couples of *Polistes simillimus*. Females were overall larger than males, but sexes also differed in body proportions, consistent with our expectation. The study generates testable hypotheses regarding the evolution of flight performance in these insects.

## 1. Introduction

Behavioral sexual roles in hymenopteran societies (ants, bees and wasps) follow a typical stereotype [1]. Females make higher investment in parental effort, building and defending the nest, and providing food to larvae. Meanwhile, males make higher investment in mating effort, as evidenced by the formation of leks and swarms. As a result, the kind and intensity of selection experienced by each sex might differ and cause dimorphism [2].

Wings are deeply involved in many biological aspects of social vespids such as nest cooling, warding-off intruders, warning potential enemies, and foraging [3]. Females can fly hundreds of meters away from the nest to capture prey and other items, to later carry significant weights back to the nest [4]. In contrast, to obtain access to a female and depending on the species, males patrol an area (individually or collectively) without defending a specific territory or actively defending territories and deter competitors [5]. Either patrolling or territorial defense might imply higher maneuverability compared to foraging females that hover in search for relatively slower prey larvae.

Sexual dimorphism in flight-related structures has seldom been addressed in these insects [6], and to our knowledge, it has never been interpreted in relation to expected flight demands. At most, there are studies comparing female body size at both interspecific and intraspecific levels. For example, wings are rounded in smaller species but elongated in larger ones [7]. Also, as the elevation increases, the body of a mountain species becomes smaller overall, while wings and mesosoma become larger [8]. Here, we describe sexual dimorphism in different body parts of a paper wasp, including those related to sex-specific flight performance (forewings, mesosoma), mating acquisition (foreleg femur), and food processing (head). We interpret such differences in relation to life history.

The neotropical *Polistes simillimus* (Zikàn,1953) was used as the model. At the end of the colony cycle the nests can contain males along with reproductive females (gynes). Around the age of one week, males permanently live the maternal colony to seek for mates, and they are not involved in parental care, which is all performed by females (De Souza, personal observation). Our results are consistent with the expectations and open a series of testable hypotheses about the influence of aerodynamics in morphological demands of wasp body size and shape.

## 2. Materials and Methods

### 2.1. Samples and Image Acquisition

**Figure 1:**
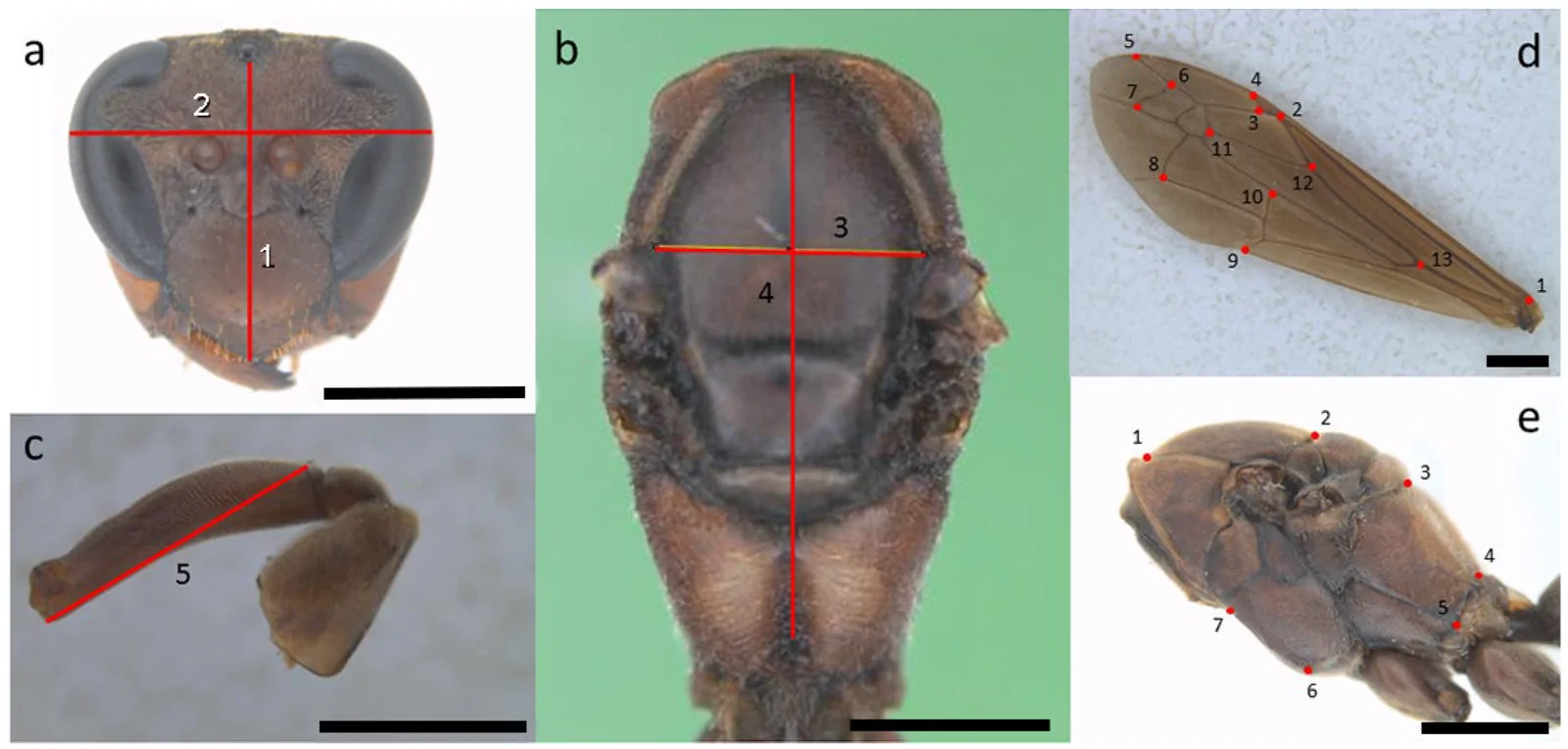
Body parts photographed. (**a**) Head, (**b**) mesosoma in dorsal view, (**c**) foreleg femur, (**d**) front wing, (**e**) mesosoma in the left lateral view. Linear measurements are indicated by the red lines: 1. head length, 2. head width, 3. mesoscutum width, 4. mesosoma length, and 5. front femur length. Landmarks are indicated by numbers and red dots in the images (**d**,**e**). Scale bar = 2 mm.

In April 2022, we sampled eleven colonies from the municipality of Muzambinho, Minas Gerais State of Brazil (21°22′ S, 46°31′ W). Wasps ($\bar{x}$ = 9, SD = 6.3 females and $\bar{x}$ = 9, SD = 8 males per colony) were frozen for 24 h and then preserved in ethanol 70% until morphological investigations were performed in 2025. Body parts were dissected and photographed under a Leica M125 C stereo microscope in conjunction with Leica Application Suite-LAS V4.12 (Leica Microsystems) software for image capture. This comprised: head in frontal view, left forewing, left foreleg femur, mesosoma in straight lateral view, and mesosoma in dorsal view (Figure 1). The magnification value for each image was adjusted according to the best framing for each structure in order to improve the accuracy of the measurements.

### 2.2. Measuring Size and Shape

Each trait we studied was measured by a single researcher. The following linear measurements were taken using ImageJ 1.53E (NIH 2008–2013) on the calibrated images (Figure 1a–c): for head: (1) height from the lower margin of median ocellus to the tip of the clypeus (head length) and (2) maximum width at higher border of toruli height (head width); for mesosoma: (3) intertegular distance (mesoscutum width) and (4) distance from the anterior border of the mesoscutum to the posterior border of the propodeum (mesosoma length); and for foreleg femur: (5) maximum oblique distance from the outer rim of the trochanter to the opposite tip of the femur (front femur length).

The shapes of the forewing and the lateral mesosoma were characterized using 13 and 7 landmarks respectively (Figure 1d,e). The number of landmarks followed the formula “2*landmarks − 4 < number of samples” (200 in our case), allowing a good representation of the structures without overfitting the analysis [9]. Wing landmarks were located at the intersections of veins covering most of the wing extension. In regard to flight, landmarks 1–5 follow the anterior border, which is the front of attack of the wing; landmarks 7–9 characterize changes at the posterior margin, where most of the drag forces are displayed during flight; and landmarks 6, 10–13 are placed at the interior area of the wing. We avoid placing landmarks at the wing border to maximize dataset completeness and to secure consistent location of each homologous point. Landmarks for mesosoma were placed at intersections of sclerites covering most of the outer border of this body part; these are located at the approximate location of the extremes of the insertion points of the wing muscles [10]. Landmarks 1, 2, 6 and 7 are associated with the indirect principal elevator muscle responsible for the upbeat of the wings, and landmarks 1, 2, 4, and 5 to the indirect and principal depressor of the muscles. The software TPSdig2 v2.31 [11] was used for landmark digitization.

The wing shape can offer information on aerodynamic trends [12]. The mesosoma shape can provide data on changes in aerodynamic characteristics and flight power [10,12]. The head and the front length femur may serve as proxies for overall body size [13].

### 2.3. Statistical Analyses

Before performing the main comparisons, outlier detection was conducted through visual inspection considering quartile distributions and the generalized Extreme Studen-tized deviate (ESD) test [14], the latter with the package cmradds [15] within R 4.5.0 [16]. Also, measurement error for mesosoma and wing shape was estimated using a Model II ANOVA based on two repeated measurements of 25 specimens for each structure [17]. Comparisons between the size of structures and their ratios across sexes were performed using Student’s *t*-tests or Welch’s *t*-tests, depending on the fulfillment of statistical assumptions. Normality was assessed using Shapiro–Wilk tests, and data were transformed when necessary, using the Box–Cox procedure to fit linear measurement data into a normal distribution [18]. Homoscedasticity was evaluated with Levene’s tests. Skewness of frequency distributions for each sex was assessed using the default functions of the package lawstat [19]. All analyses were conducted in R 124 4.5.0 [16].

Scaling relationships between the size of structures were investigated using the package smatr 3.4–8 [20] in R 4.5.0 [16]. All variables were log10-transformed prior to analyses. Standard Major Axis (SMA) regressions were fitted using robust Huber’s M estimation to reduce the influence of outliers [21]. For each SMA model, residual normality was assessed with Shapiro–Wilk tests, and homoscedasticity was evaluated through visual inspection of residual-versus-fitted plots [20]. Deviations from isometry were tested separately for each variable within each sex. Differences in SMA slopes and elevations between sexes were also evaluated.

Sex differences in size and shape were also analyzed using geometric morphometric methods based on a full Procrustes fit. For both forewing and mesosoma datasets, Principal Component Analysis (PCA), Procrustes MANOVA, Discriminant Function Analysis (DFA), and allometric regressions were performed. These analyses were conducted using MorphoJ [22] and smatr 3.4–8 [20], with R 4.5.0 [16] used for data processing and statistical analyses. In all analyses, the alpha level was set at α = 0.05.

## 3. Results

### 3.1. Outliers, Transformations, and Measurement Error

No outliers were recorded for any of the linear variables (Table S1), nor the forewing or mesosoma shape (Figure S1). The Procrustes ANOVA tests for centroid size and for shape of both mesosoma and forewing indicated reliability of the measurements as in all cases the interindividual variance was larger than the intraindividual variance (error) (Table 1).

### 3.2. Sex Differences in Size and Shape

**Figure 2:**
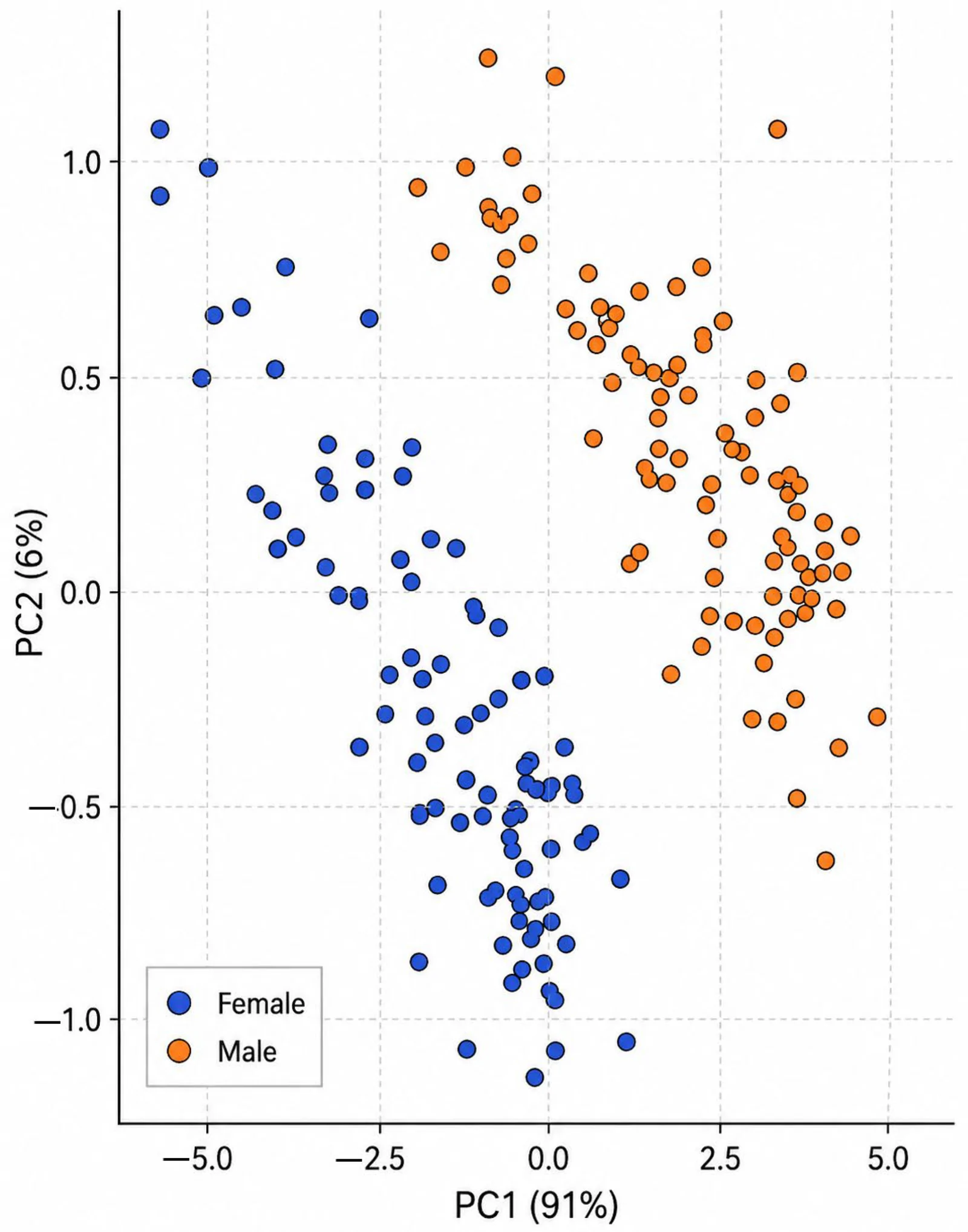
Principal Component Analysis of the linear measurements for each sex.

**Figure 3:**
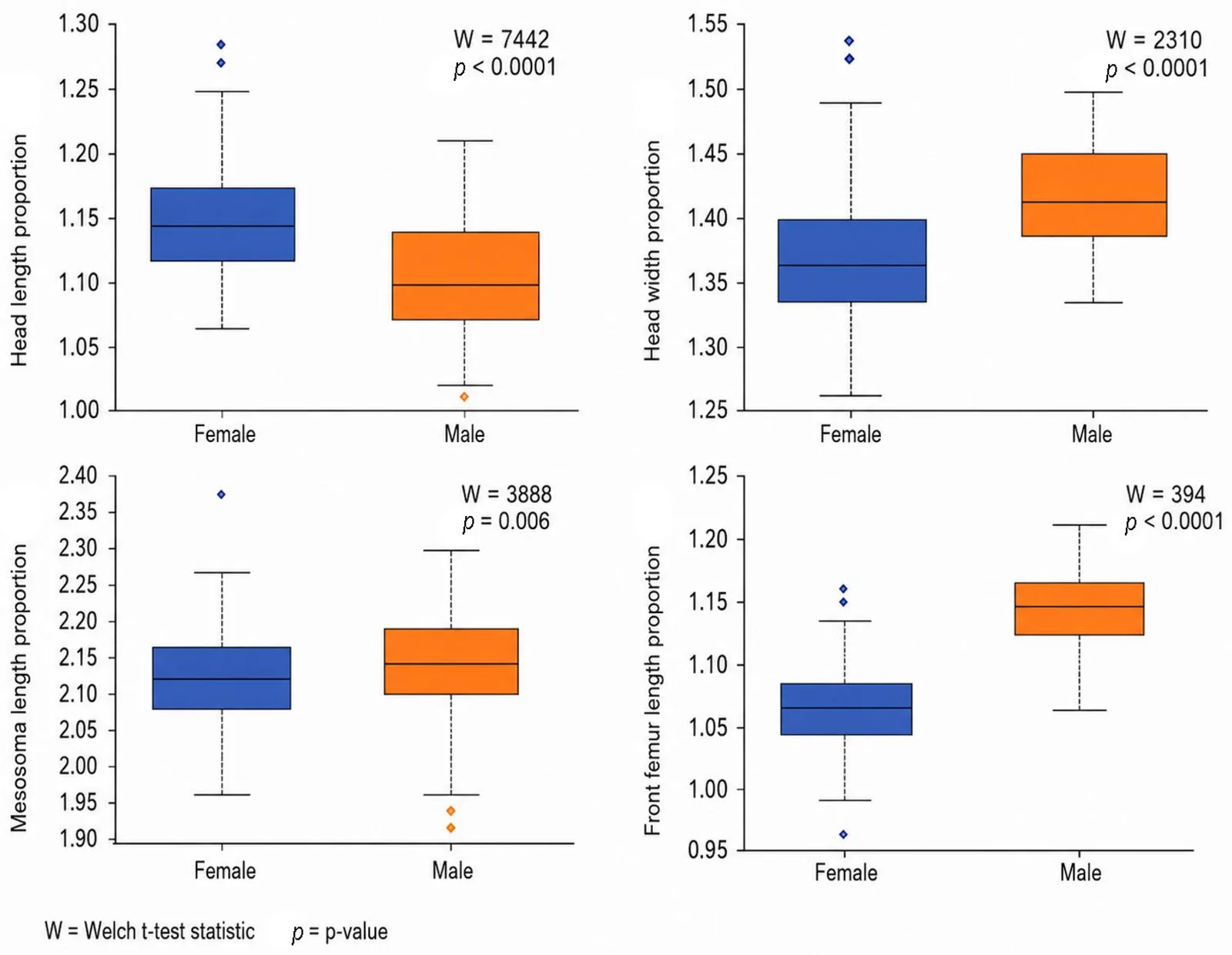
Different linear measurements corrected by mesoscutum width across sexes.

Females were statistically larger than males in all measurements (Table 2). Instead, skewness in linear measurements depended on the sex and the body part examined (Table 3). Sexes were clearly differentiated in linear measurements by PCA (Figure 2). The mesoscutum width was the variable that most approximates the first component of the PCA and thus the best descriptor of body size. The ratios between the mesoscutum width and the other linear measurements also showed differences between sexes. Specifically, males have disproportionally smaller head length, but larger head width, mesosoma length, and front femur length than females (Figure 3).

**Figure 4:**
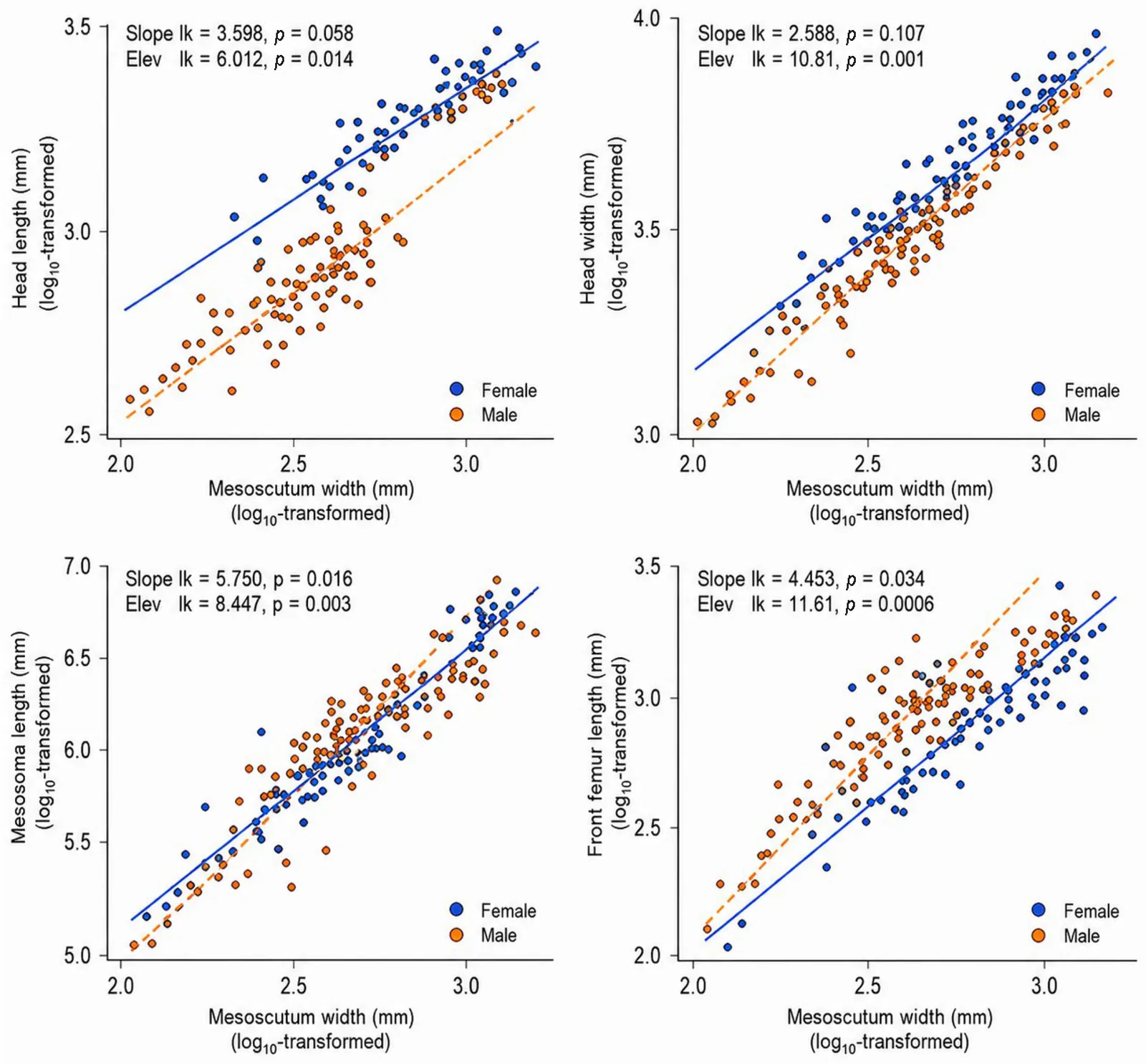
Scaling relationships across sexes.

As depicted in Figure 4 and Table 4, mesosoma length was positively allometric in males but isometric in females. Front femur length was negatively allometric in females but isometric in males. Head measurements were negatively allometric in both sexes. As expected, there were significant differences in the elevation of the allometric regressions for all structures, with females being larger in all cases. All structures show a steeper slope for males, although statistical differences between sexes were identified for mesosoma length and front femur length only.

**Figure 5:**
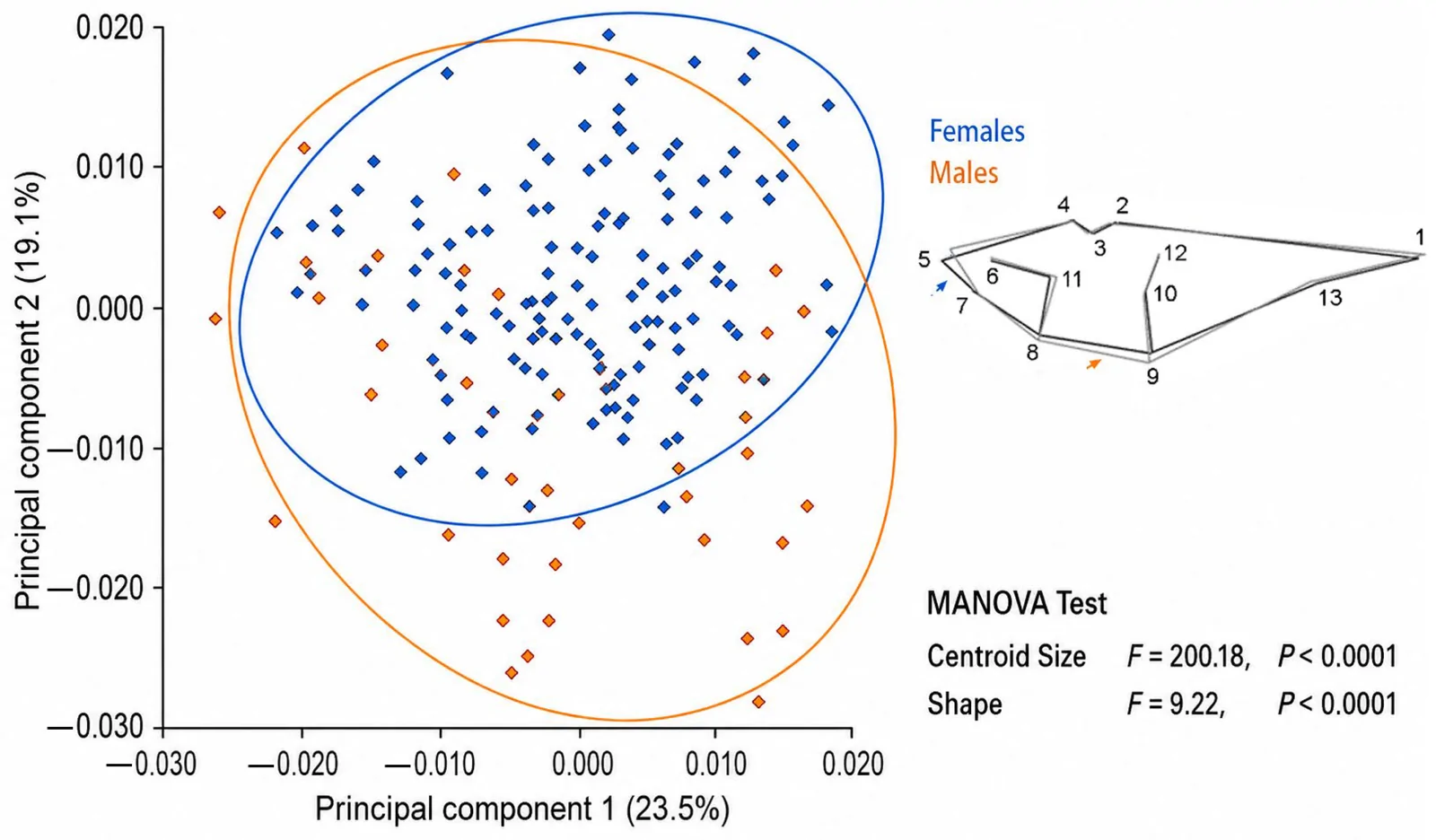
Principal Component Analysis of forewing shape across sexes, with landmarks and configuration changes indicated on the right. Arrows point to the shape of each sex following the color codes, numbers refer to landmarks as described in the methods.

**Figure 6:**
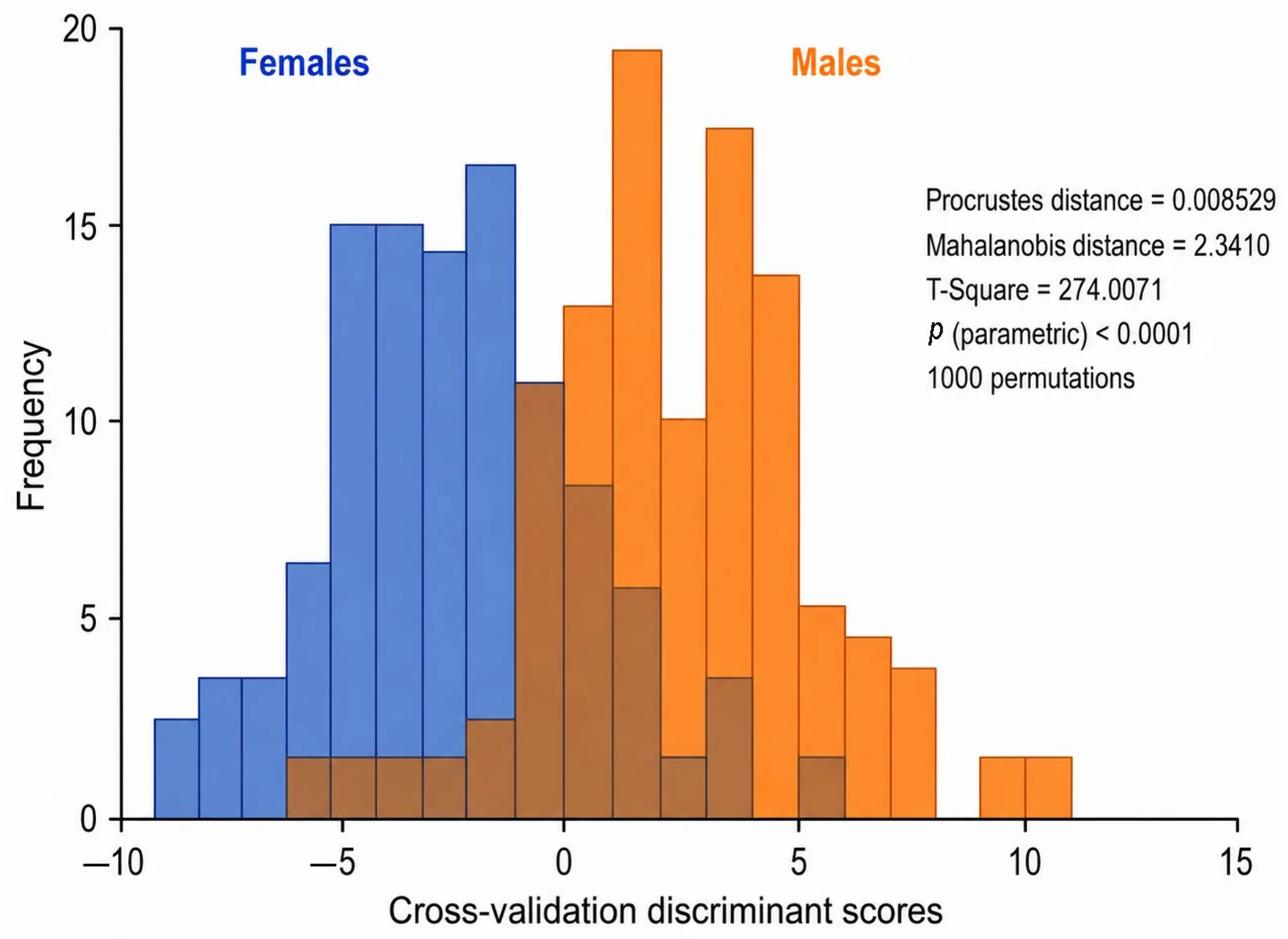
Discriminant Function Analysis of forewing shape across sexes.

Forewing size and shape differed across sexes (Procrustes MANOVA *p* < 0.0001, Discriminant Function Analysis *t* = 274, *p* < 0.0001, Figure 5 and Figure 6). As observed by the displacement backwards of landmarks 5, 8, and 9, males exhibit rounder wings compared to females (Figure 5).

**Figure 7:**
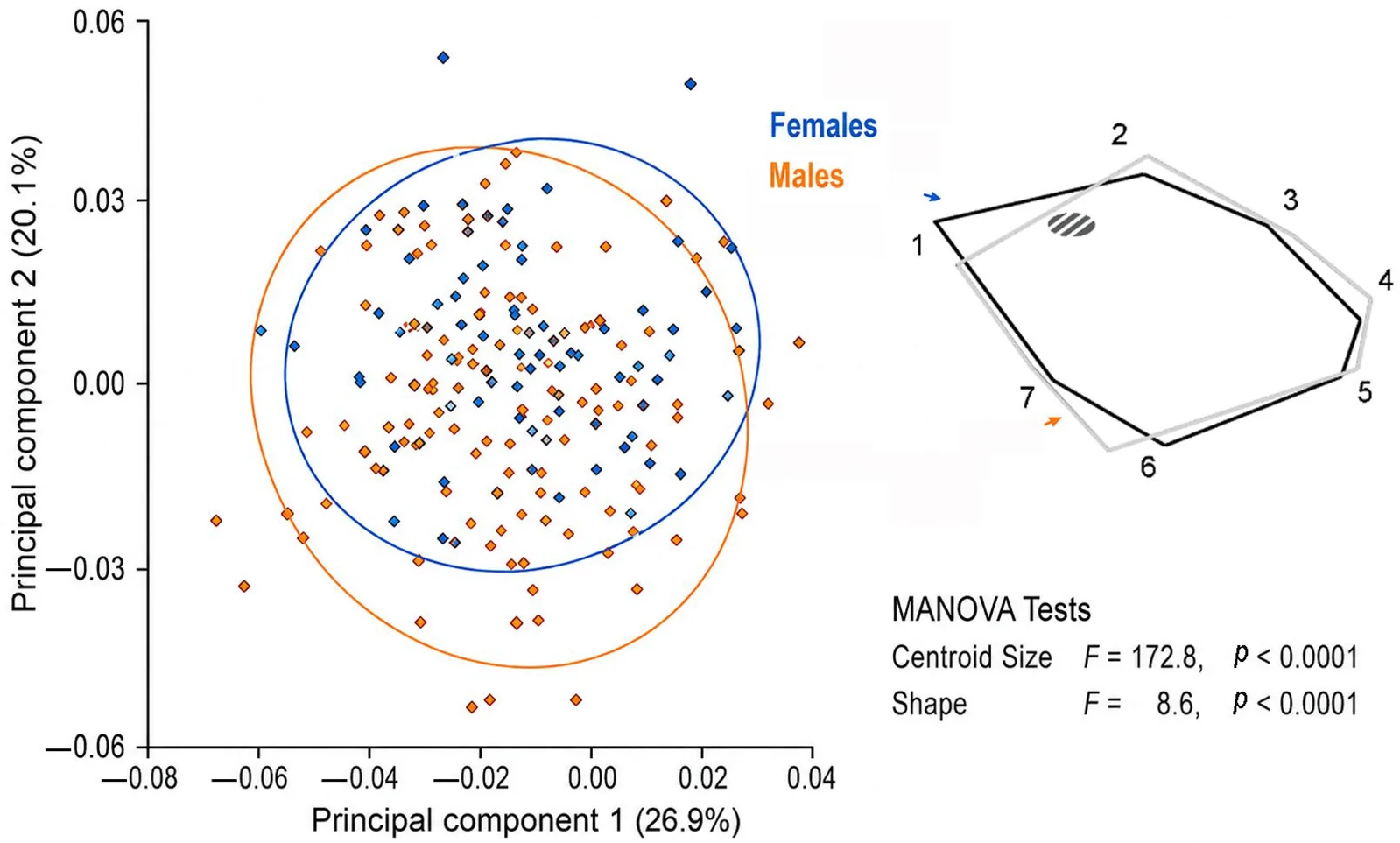
Principal Component Analysis of mesosoma across sexes, with landmarks and configuration changes indicated on the right. The dashed oval on the configuration indicates the location of the wing insertion area. Arrows point to mesosoma configurations by sex. Numbers are landmarks as described in methods.

**Figure 8:**
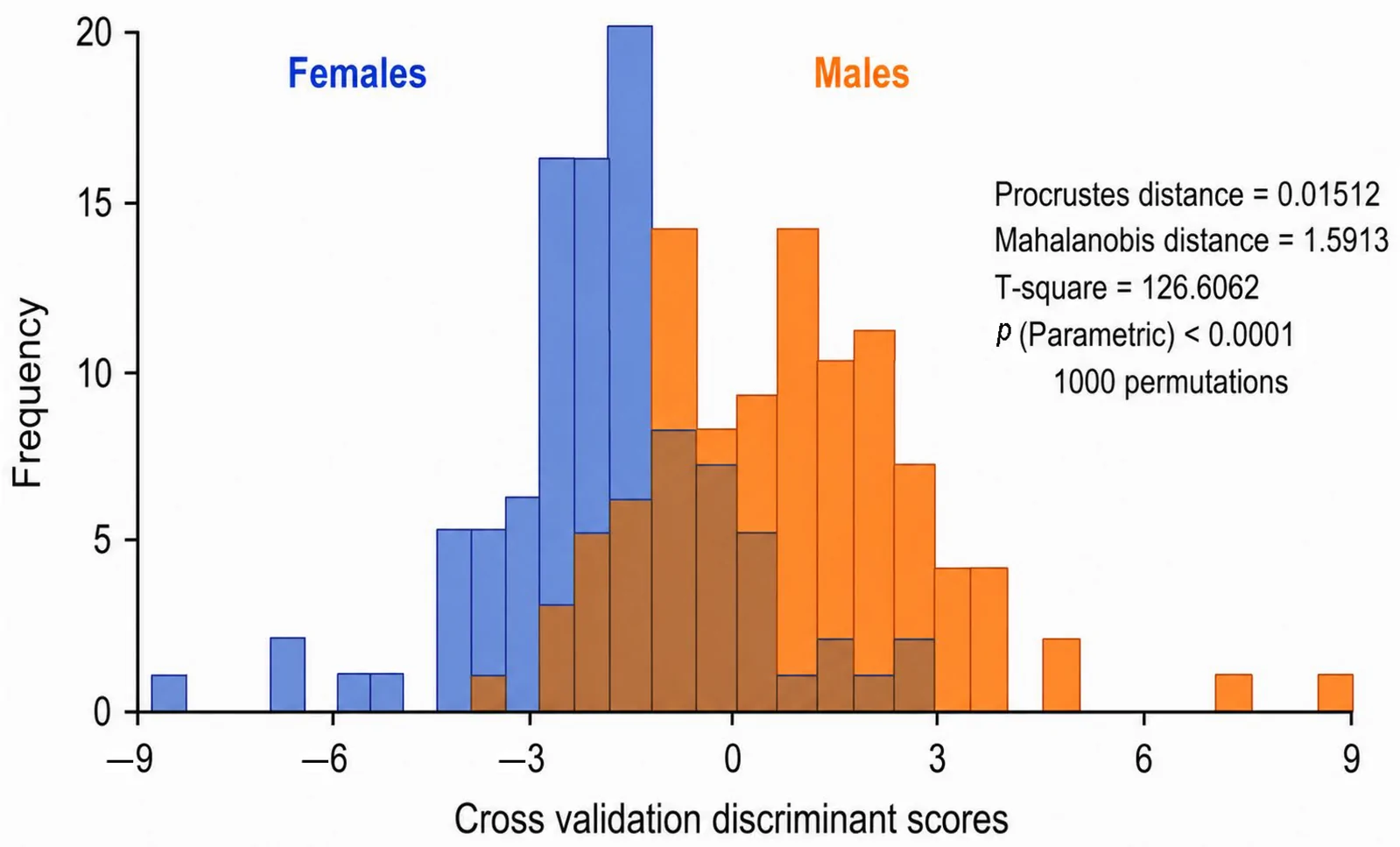
Discriminant Function Analysis of mesosoma shape across sexes.

The size and shape of the mesosoma also differed between sexes (Procrustes MANOVA *p* < 0.0001, Discriminant Function Analysis *t* = 126.6, *p* < 0.0001, Figure 7 and Figure 8). Males exhibited a stouter mesosoma due a higher dorsoventral axis and a shorter anteroposterior axis. The higher mesosoma in males is a consequence of the displacement of landmark 2 upwards, and landmarks 6 and 7 downwards. The lengthening of the mesosoma is mainly a consequence of a strong anterior displacement of landmark 1.

**Figure 9:**
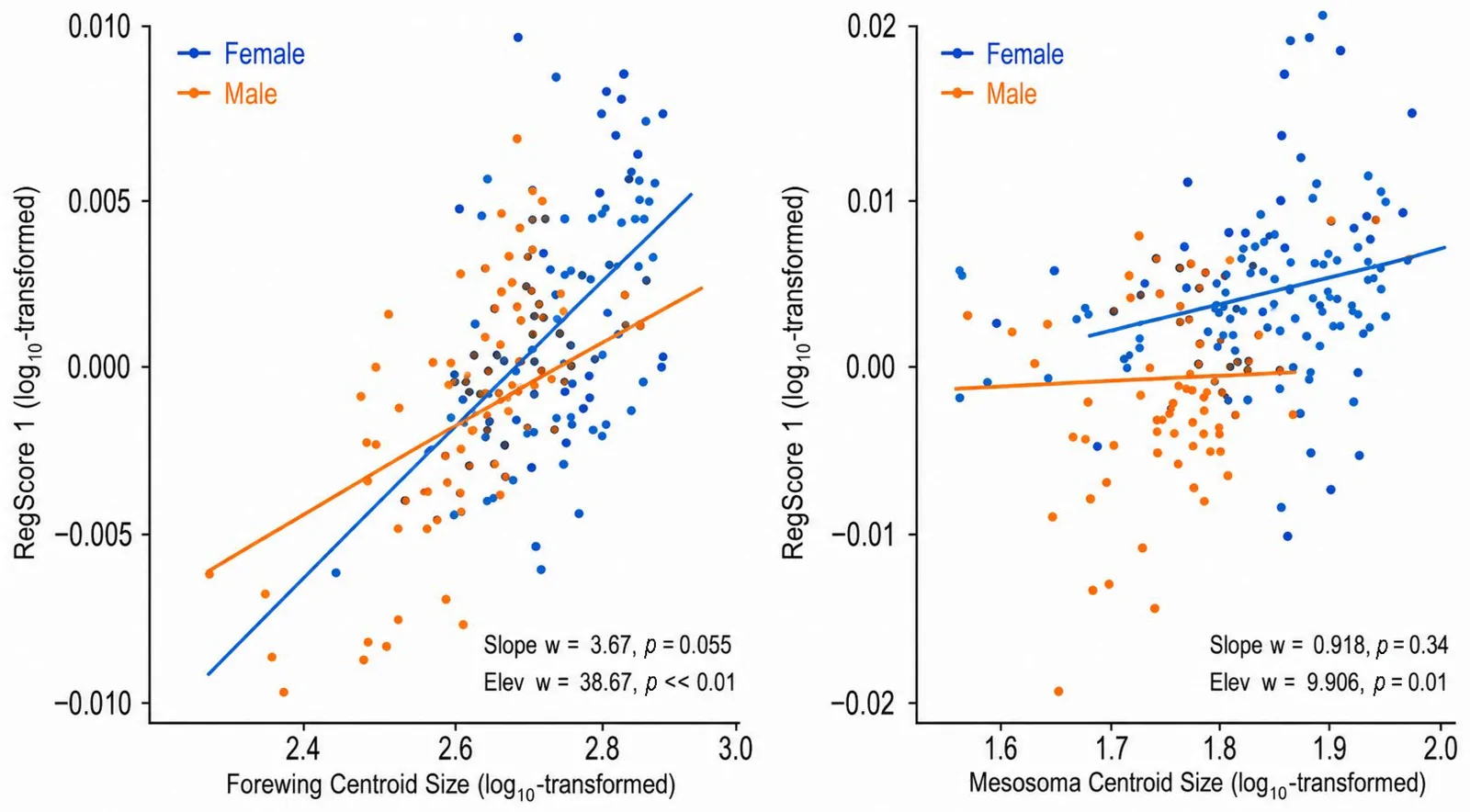
Regressions across sexes for forewing and mesosoma centroid sizes.

Regression analyses between the centroid size of the configurations and Procrustes coordinates for the forewing and the mesosoma confirmed differentiation in the elevation between sexes although the slopes remain similar. The percentage of variation explained by the regressions was only 7.7% for the wings and 2.6% for mesosoma (Figure 9).

## 4. Discussion

Females were consistently larger than males in all linear measurements. The sexes also differed in body proportions, allometric trajectories, and the shape of body parts.

Females in social hymenopterans perform virtually all parental care, including nest construction, prey capture, transportation of water and plant fibers, brood provisioning, and colony defense [23]. These activities might favor larger body size, which increases carrying capacity and prey-handling ability. In addition, female social insects are under consistent selection for higher fertility and thus large abdomen size [24], and males are generally unable to monopolize groups of females, making rare species in which males are larger than females [25]. Among social wasps, direct competition for reproductive opportunities may impose sexual selection on males [26]. However, the direction of selection on body size is not universal in insects. Depending on the mating system, territorial behavior, and ecological context, sexual selection may favor either larger males through increased fighting ability or smaller males through enhanced maneuverability or alternative reproductive tactics [27,28]. In *P. simillimus*, the larger size of females likely reflects the stronger influence of parental care and fertility demands [29,30,31].

Sex differences remained evident after correcting for body size. Accordingly, the head was wider in males and longer in females, and males had longer fore femora and mesosomas. The female feeding might involve intense mandible activity (biting, holding and macerating), which may have contributed to the observed dimorphism. A longer fore femora may facilitate grasping females during copulation [5] and a longer mesosoma may increase indirect flight muscles and boost flight performance related to male–male competition [32].

The shape of the mesosoma of males is comparatively stouter in the anteroposterior axis and higher in the dorsoventral axis. These changes move forward the center of the body mass of males relative to the location of the wing insertion and reduce the distance between wing base and body mass center. These modifications can increase aerodynamic stability and responsiveness to dynamic torque [12,33]; additionally, they suggest a larger indirect principal elevator muscle involved in upbeat movement of the wings and thus vertical displacement. Larger male wings are also consistent with a larger elevator muscle as more force is required to upbeat the wing. These modifications may not be the only ones responsible for a more maneuverable flight as other structures and behaviors can be involved, such as steering muscles, legs, metasoma, and asymmetric flapping [12,34]. These findings further suggest that sex dimorphism evolved as a result of contrasting mating and parental demands experienced by males and females.

The observed differences in wing shape reinforce this interpretation. Rounded wings observed in males generally improve maneuverability, whereas elongated wings observed in females reduce the energetic cost of sustained flight. Although these aerodynamic relationships have been studied and proposed primarily in birds [35] and a limited number of insects [7,36], they provide a plausible functional framework for interpreting our results. Male *Polistes* patrol mating areas, pursue rivals, and perform rapid directional changes during reproductive interactions [26,37], whereas females repeatedly commute between the nest and foraging sites while transporting prey, water, or nest-building material [23].

The allometric analyses further suggest that males and females follow distinct growth trajectories. Mesosoma length exhibited positive allometry in males but remained isometric in females, whereas front femur length exhibited negative allometry in females but remained isometric in males. These sex-specific scaling patterns indicate that different body structures are preferentially modified with increasing body size in each sex.

Our study generates several testable hypotheses regarding the functional significance of these morphological differences and developmental patterns. Flight performance experiments could determine whether males indeed exhibit greater maneuverability and acceleration, whereas females achieve higher energetic efficiency during sustained flight. Likewise, micro-computed tomography or direct measurements of flight-muscle volume could test whether the relatively deeper mesosoma of males actually contains proportionally larger indirect flight muscles. Such studies would establish a direct link between morphology, biomechanics, development, and the divergent behavioral roles of males and females in social wasps.

## Figures and Tables

**Table 1:** Measurement error.

Structure	Interindividual Variance	Intraindividual Variance (Error)
Mesosoma centroid size	0.502417	0.001516
Mesosoma shape	0.000204	0.000081
Forewing centroid size	2.064638	0.001722
Forewing shape	0.000029	0.000003

**Table 2:** Sex differences in body size and shape.

Variable	Females	Males	*t*-Tests
Head length (mm)	3.254 ± SD = 0.136 (Range = 3.521–2.851)	2.706 ± SD = 0.129 (Range = 2.957–2.375)	*t* = 29.21, df = 198, *p* < 0.001
Head width (mm)	3.923 ± SD = 0.166 (Range = 4.250–3.516)	3.513 ± SD = 0.172 (Range = 3.820–3.072)	*t* = 17.07, df = 198, *p* < 0.001
Mesosoma length (mm)	5.988 ± SD = 0.404 (Range = 6.739–5.045)	5.234 ± SD = 0.408 (Range = 6.032–4.221)	*t* = 13.13, df = 198, *p* < 0.001
Mesoscutum width (mm)	2.856 ± SD = 0.207 (Range = 3.231–2.288)	2.465 ± SD = 0.173 (Range = 2.773–2.020)	*t* = 14.49, df = 198, *p* < 0.001
Front femur length (mm)	2.954 ± SD = 0.157 (Range = 3.264–2.593)	2.802 ± SD = 0.180 (Range = 3.114–2.278)	*t* = 6.34, df = 198, *p* < 0.001
Wing shape (centroid size)	15.30 ± SD = 0.935 (Range = 16.80–13.07)	13.333 ± SD = 1.030 (Range = 15.0–10.14)	*t* = 14.151, df = 198, *p* < 0.001
Mesosoma shape (centroid size)	5.938 ± SD = 0.545 (Range = 7.074–4.607)	6.353 ± SD = 0.604 (Range = 7.402–4.607)	*t* = 13.15, df = 198, *p* < 0.001

**Table 3:** Skewness in body size across sexes.

Variable	Skewness	
	Females	Males
Head length (mm)	−1.53, *p* = 0.126	−1.87, *p* = 0.062
Head width (mm)	−2.41, *p* = 0.03	−3.13, *p* = 0.004
Mesosoma length (mm)	−2.26, *p* = 0.034	−3.29, *p* = 0.002
Mesoscutum width (mm)	−2.26, *p* = 0.064	−3.29, *p* = 0.0001
Front femur length (mm)	−0.42, *p* = 0.7	−3.57, *p* = 0.0001

**Table 4:** Allometric differences across sexes.

Structure	Females				Males				Slope Comparison (lk, *p*)	Elevation Comparison (lk, *p*)
	Slope	R^2^	*t*	*p*	Slope	R^2^	*t*	*p*		
Head length	0.570	0.84	0.838	<0.0001	0.675	0.70	−0.594	<0.0001	3.598, 0.058	6.012, 0.014
Head width	0.575	0.82	−0.825	<0.0001	0.697	0.83	−0.669	<0.0001	2.588, 0.107	10.81, 0.001
Mesosoma length	0.917	0.80	−0.189	0.059	1.114	0.77	0.222	0.026	5.750, 0.016	8.447, 0.003
Front femur length	0.725	0.77	−0.562	<0.0001	0.924	0.75	−0.158	0.116	4.453, 0.034	11.61, 0.0006

## Data Availability

All data used in the study are available as Supplementary Files.

## Supplementary Materials

The following supporting information can be downloaded at https://www.mdpi.com/article/10.3390/insects17080794/s1.
