# Supplementary material for: Sexual Dimorphism in Flight-Related Structures of a Paper Wasp
Source: Insects. 2026 Jul 31;17(8):794. doi: 10.3390/insects17080794 (PMC13513388; doi:10.3390/insects17080794)
Supplement: Supplementary file 1 [file insects-17-00794-s001.zip › Updated Supplementary material stats outputs.pdf]

SUPPLEMENTARY MATERIAL

Figure S1. MorphoJ analysis of outlier detection. Screenshots of MorphoJ showing no outliers for forewing and mesosoma configurations, as there were no individuals significantly departing from the theoretical Mahalanobis distance.

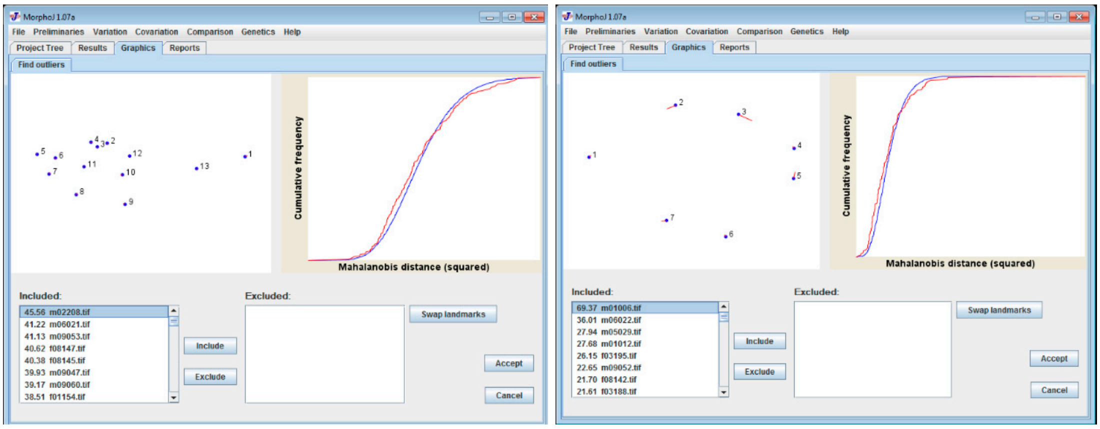

Table S1. ESD tests. Output of the outlier identification for each linear measurement and each sex. The first 6 individuals with the extreme values are tested. A False result means no outlier.

ESD test output

ESD_test(female head length, alpha = 0.05, h = 5)

$stat

	i	Mean	SD	x	Obs	ESDi	Lambda	Outlier
1	1	3.254460	0.1359191	2.851	1	2.968383	3.384083	FALSE
2	2	3.258535	0.1303254	2.924	2	2.566923	3.380651	FALSE
3	3	3.261949	0.1264686	2.957	4	2.411263	3.377176	FALSE
4	4	3.265093	0.1232162	2.981	5	2.305645	3.373658	FALSE
5	5	3.268052	0.1203479	3.004	11	2.194072	3.370097	FALSE
6	6	3.270832	0.1178480	3.016	13	2.162374	3.366490	FALSE

> ESD_test(female head width, alpha = 0.05, h = 5)

$stat

	i	Mean	SD	x	Obs	ESDi	Lambda	Outlier
1	1	3.923270	0.1662897	3.516	1	2.449159	3.384083	FALSE
2	2	3.927384	0.1619408	3.552	2	2.318032	3.380651	FALSE
3	3	3.931214	0.1582012	3.608	3	2.043058	3.377176	FALSE
4	4	3.934546	0.1555279	4.250	93	2.028277	3.373658	FALSE
5	5	3.931260	0.1529220	3.622	4	2.022341	3.370097	FALSE
6	6	3.934516	0.1503521	3.641	9	1.952190	3.366490	FALSE

```
> ESD_test(female mesosoma length, alpha = 0.05, h = 5)
$stat
  i    Mean       SD     x Obs    ESDi   Lambda Outlier
1 1 5.988500 0.4048979 5.045   7 2.330217 3.384083   FALSE
2 2 5.998030 0.3955248 5.097   2 2.278063 3.380651   FALSE
3 3 6.007224 0.3867785 5.226  13 2.019824 3.377176   FALSE
4 4 6.015278 0.3804378 5.288   5 1.911688 3.373658   FALSE
5 5 6.022854 0.3750076 5.298  14 1.932905 3.370097   FALSE
6 6 6.030484 0.3694299 6.739  99 1.917863 3.366490   FALSE
```

```
> ESD_test(female mesoscutum width, alpha = 0.05, h = 5)
$stat
  i    Mean       SD     x Obs    ESDi   Lambda Outlier
1 1 2.856540 0.2072201 2.288   2 2.743653 3.384083   FALSE
2 2 2.862283 0.2001167 2.341   1 2.604895 3.380651   FALSE
3 3 2.867602 0.1939834 2.355   7 2.642505 3.377176   FALSE
4 4 2.872887 0.1877664 2.481  14 2.087097 3.373658   FALSE
5 5 2.876969 0.1843744 2.499   3 2.050007 3.370097   FALSE
6 6 2.880947 0.1811622 2.516   9 2.014478 3.366490   FALSE
```

```
> ESD_test(female front femur, alpha = 0.05, h = 5)
$stat
  i    Mean       SD     x Obs    ESDi   Lambda Outlier
1 1 2.954420 0.1571692 2.593   1 2.299561 3.384083   FALSE
2 2 2.958071 0.1536484 2.604   2 2.304421 3.380651   FALSE
3 3 2.961684 0.1501519 2.617   3 2.295566 3.377176   FALSE
4 4 2.965237 0.1467315 2.654   4 2.121134 3.373658   FALSE
5 5 2.968479 0.1439669 2.665   5 2.107979 3.370097   FALSE
6 6 2.971674 0.1412688 2.671   6 2.128381 3.366490   FALSE
```

```
> ESD_test(male head length, alpha = 0.05, h = 5)
$stat
  i    Mean       SD     x Obs    ESDi   Lambda Outlier
1 1 2.705770 0.1296469 2.375   1 2.551314 3.384083   FALSE
2 2 2.709111 0.1259053 2.397   2 2.478936 3.380651   FALSE
3 3 2.712296 0.1224788 2.420   3 2.386502 3.377176   FALSE
4 4 2.715309 0.1194076 2.425   4 2.431246 3.373658   FALSE
5 5 2.718333 0.1162406 2.433   5 2.454680 3.370097   FALSE
6 6 2.721337 0.1130503 2.447   6 2.426679 3.366490   FALSE
```

```
> ESD_test(male head width, alpha = 0.05, h = 5)
$stat
  i    Mean       SD     x Obs    ESDi   Lambda Outlier
1 1 3.513960 0.1727498 3.072   3 2.558383 3.384083   FALSE
```

```
2 2 3.518424 0.1677311 3.073   2 2.655586 3.380651   FALSE
3 3 3.522969 0.1623499 3.095   1 2.636093 3.377176   FALSE
4 4 3.527381 0.1571766 3.131   7 2.521886 3.373658   FALSE
5 5 3.531510 0.1526219 3.139   5 2.571784 3.370097   FALSE
6 6 3.535642 0.1479358 3.145   6 2.640619 3.366490   FALSE
```

```
> ESD_test(male mesosoma length, alpha = 0.05, h = 5)
$stat
  i     Mean        SD     x Obs     ESDi   Lambda Outlier
1 1 5.233950 0.4079285 4.221   2 2.483156 3.384083   FALSE
2 2 5.244182 0.3968978 4.222   1 2.575428 3.380651   FALSE
3 3 5.254612 0.3850587 4.339   7 2.377851 3.377176   FALSE
4 4 5.264052 0.3754889 4.374   3 2.370381 3.373658   FALSE
5 5 5.273323 0.3661288 4.430   6 2.303350 3.370097   FALSE
6 6 5.282200 0.3575344 4.512   8 2.154198 3.366490   FALSE
```

```
> ESD_test(male mesoscutum width, alpha = 0.05, h = 5)
$stat
  i     Mean        SD     x Obs     ESDi   Lambda Outlier
1 1 2.465600 0.1727584 2.020   2 2.579324 3.384083   FALSE
2 2 2.470101 0.1676408 2.050   3 2.505959 3.380651   FALSE
3 3 2.474388 0.1629576 2.061   1 2.536782 3.377176   FALSE
4 4 2.478649 0.1582193 2.096   6 2.418476 3.373658   FALSE
5 5 2.482635 0.1540763 2.098   7 2.496395 3.370097   FALSE
6 6 2.486684 0.1496717 2.129   8 2.389792 3.366490   FALSE
```

```
> ESD_test(male front femur, alpha = 0.05, h = 5)
$stat
  i     Mean        SD     x Obs     ESDi   Lambda Outlier
1 1 2.802600 0.1805477 2.278   1 2.905604 3.384083   FALSE
2 2 2.807899 0.1734748 2.317   2 2.829800 3.380651   FALSE
3 3 2.812908 0.1670151 2.335   6 2.861467 3.377176   FALSE
4 4 2.817835 0.1605645 2.417   7 2.496412 3.373658   FALSE
5 5 2.822010 0.1560239 2.423   3 2.557367 3.370097   FALSE
6 6 2.826211 0.1512974 2.431   8 2.612144 3.366490   FALSE
```

Table S2. Transformations, normality, and homoscedasticity tests. Variables that do not fit requirements after transformation were tested with non-parametric analyses.

	Box-Cox Transformation tests for normality			
Variable	Females		Males	
	Lambda	Normality Shapiro test	lambda	Normality Shapiro test
Head_length	6.7	W = 0.99031, p = 0.69	6.2	W = 0.98777, p = 0.49
Head_width	4.7	W = 0.98318, p = 0.23	8.3	W = 0.98502, p = 0.32

Mesosoma_length	1.8	W = 0.97523, p = 0.05	4.7	W = 0.98478, p = 0.30
Mesoscutum_width	4.3	W = 0.97497, p = 0.05	5.1	W = 0.98329, p = 0.24
Front_femur_length	4.6	W = 0.98206, p = 0.19	7.1	W = 0.98078, p = 0.15

Variable	Levene´s test for homocesdasticity
Head_length	MSq= 0.000739, F= 0.118, P= 0.73
Head_width	MSq= 0.000207, F= 0.021, P= 0.88
Mesosoma_length	MSq= 0.00038, F= 0.007, P= 0.93
Mesoscutum_width	MSq= 0.06220, F= 5.475, P= 0.02*
Front_femur_length	MSq= 0.014215, F= 1.444, P= 0.23

Table S3. Linear measurements and centroid size datasets
Available as excel file “Measurements 3.xls”

Table S4.TPS: configurations of mesosoma. Available as tps file “configurations of mesosoma.TPS”

Table S5.TPS: configurations of wings. Available as tps file “configurations of wing.TPS”
